# Prevalence of malaria infection in pregnant women compared with children for tracking malaria transmission in sub-Saharan Africa: a systematic review and meta-analysis

**DOI:** 10.1016/S2214-109X(15)00049-2

**Published:** 2015-08-19

**Authors:** Anna M van Eijk, Jenny Hill, Abdisalan M Noor, Robert W Snow, Feiko O ter Kuile

**Affiliations:** a Department of Clinical Sciences, Liverpool School of Tropical Medicine, Liverpool, UK; b Spatial Health Metrics Group, Department of Public Health Research, KEMRI-Wellcome Trust Research Program, Nairobi, Kenya; c Centre for Tropical Medicine & Global Health, Nuffield Department of Clinical Medicine, University of Oxford, Centre for Clinical Vaccinology and Tropical Medicine, Oxford, UK

## Abstract

**Background:**

In malarious areas, pregnant women are more likely to have detectable malaria than are their non-pregnant peers, and the excess risk of infection varies with gravidity. Pregnant women attending antenatal clinic for their first visit are a potential pragmatic sentinel group to track the intensity of malaria transmission; however, the relation between malaria prevalence in children, a standard measure to estimate malaria endemicity, and pregnant women has never been compared.

**Methods:**

We obtained data on malaria prevalence in pregnancy from the Malaria in Pregnancy Library (January, 2015) and data for children (0–59 months) were obtained from recently published work on parasite prevalence in Africa and the Malaria in Pregnancy Library. We used random effects meta-analysis to obtain a pooled prevalence ratio (PPR) of malaria in children versus pregnant women (during pregnancy, not at delivery) and by gravidity, and we used meta-regression to assess factors affecting the prevalence ratio.

**Findings:**

We used data from 18 sources that included 57 data points. There was a strong linear relation between the prevalence of malaria infection in pregnant women and children (r=0·87, p<0·0001). Prevalence was higher in children when compared with all gravidae (PPR=1·44, 95% CI 1·29–1·62; *I*^2^=80%, 57 studies), and against multigravidae (1·94, 1·68–2·24; *I*^2^=80%, 7 studies), and marginally higher against primigravidae (1·16, 1·05–1·29; *I*^2^=48%, 8 studies). PPR was higher in areas of higher transmission.

**Interpretation:**

Malaria prevalence in pregnant women is strongly correlated with prevalence data in children obtained from household surveys, and could provide a pragmatic adjunct to survey strategies to track trends in malaria transmission in Africa.

**Funding:**

The Malaria in Pregnancy Consortium, which is funded through a grant from the Bill & Melinda Gates Foundation to the Liverpool School of Tropical Medicine, UK; US Centers for Disease Control and Prevention; and Wellcome Trust, UK.

## Introduction

In malaria transmission areas, pregnant women—in particular primigravidae—are known to be susceptible to malaria and to have higher prevalence and densities of parasitaemia than are non-pregnant women from the same population.^1^ The size of the excess risk varies with the age of the pregnant woman, reflecting cumulative exposure to malaria over a lifetime, and with parity, as a result of pregnancy-specific immunity acquired after exposure to malaria in previous pregnancies. The consequences of malaria infection during pregnancy will depend on maternal malaria immune status; however, infections are associated with maternal anaemia and fetal growth retardation, and can result in acute illness, pregnancy loss or preterm delivery, and even maternal mortality.

The World Health Organization recommends use of insecticide-treated nets (ITNs) and intermittent preventive treatment in pregnancy (IPTp) with a dose of sulfadoxine-pyrimethamine at every scheduled antenatal care visit for the prevention of malaria in pregnancy in areas with moderate-to-high malaria transmission.^2^, ^3^, ^4^ However, because of rising parasite resistance to sulfadoxine-pyrimethamine and decreasing malaria transmission in some regions, alternative strategies for IPTp are now being assessed, such as screening and treatment strategies in pregnancy. This approach consists of the use of rapid diagnostic tests to screen women for malaria at the first or each antenatal visit and treatment of positive women with artemisinin combination therapies.^5^

Data for malaria prevalence in children obtained from household surveys, such as malaria indicator surveys or school-based surveys, are used to measure transmission intensity and success of malaria control activities in a region.^6^, ^7^ Household surveys are logistically demanding and expensive. School surveys, by contrast, are cheaper to do and often include larger sampled populations;^8^ however, neither approach provides a simple routine real-time measure of malaria in the community. Pregnant women attending antenatal care are a potential alternative source of data for malaria prevalence.

A systematic review^9^ showed that antenatal clinic attendance in pregnant women in most countries in sub-Saharan Africa is high, with at least 75% of pregnant women attending one or more visits in 44 countries in 2010, and at least 90% of pregnant women doing so in 21 countries. That pregnant women are easily accessible for contact at antenatal clinics especially for first visits, makes them a potential surveillance population to track malaria transmission intensity. Because women at the first antenatal clinic visit have not yet received their first dose of sulfadoxine-pyrimethamine for IPTp, malaria infection prevalence at this first visit is likely to be an indicator of malaria transmission intensity in their community. Information on the prevalence of malaria infection at the antenatal booking appointment may become more widely available if screen and treat approaches for malaria control in pregnant women were to be adopted in areas with low or reduced transmission in Africa.^5^

In this meta-analysis, we investigate the relation between malaria infection prevalence in pregnant women and the more standard reference population of children from the same community. We use assembled data from across Africa published since 1983 to assess how any correlation might be modified by gravidity and malaria transmission intensity.^10^

## Methods

### Search strategy and selection criteria

We obtained data on the prevalence of malaria infection in pregnant women from the Malaria in Pregnancy Library.^11^ This library is a comprehensive bibliographic database created by the Malaria in Pregnancy Consortium that is updated every 4 months with a standardised protocol to search more than 40 sources, including PubMed, Web of Knowledge, and Google Scholar.^12^ We used data up to January, 2015, without language restriction.^12^

Inclusion criteria were: studies in sub-Saharan Africa, based in either the community or antenatal clinics, that screened pregnant women for malaria parasitaemia by microscopy or rapid diagnostic test, irrespective of the presence of symptoms. We excluded studies that selected only women with a history of fever or malaria, and studies that diagnosed malaria at delivery, so that the data for pregnant women would be comparable with those for women attending antenatal clinic. There was no time limit for inclusion and we did not restrict study selection to those with first antenatal visit data.

We undertook a systematic evaluation of studies in pregnant women and extracted data including study location, year of study, study population, inclusion and exclusion criteria used, use of malaria prevention strategies (ITNs, IPTp, or prophylaxis), type of malaria diagnostic test used, and test results. Where sufficient information was available, data were extracted by gravidity group, study site, and malaria season. Where needed, and if possible, we contacted authors of the included studies for additional information.

Data on the prevalence of malaria infection in pregnant women were then selected on the basis of the availability of the same prevalence data in children aged 0–59 months collected during the same study period and in the same locality as the data in pregnant women. The contemporaneous prevalence data in children and pregnant women were either extracted from studies reported in the Malaria in Pregnancy Library that also reported data in children, or obtained from surveys that collected data on pregnant women and children simultaneously. We identified these data from the large database of over 28 483 temporally and spatially unique surveys of malaria infection undertaken across Africa since 1980 and described elsewhere,^6^ and from nationally representative household surveys, such as Demographic and Health Surveys, Multiple Indicator Cluster Surveys, and Malaria Indicator Surveys.^13^, ^14^, ^15^ An overview of the methods used in these surveys has been reported previously.^9^, ^16^ The information we extracted from the child records included study population, inclusion and exclusion criteria used, use of ITNs, type of malaria diagnostic test used, and test results.

We assessed the quality of studies after considering source population, participant selection, appropriate tests, characteristics reporting, and completeness of outcome data. Quality was classified as low-to-moderate or good. Further details of the methods used to assess quality are included in the appendix.

### Statistical analysis

Meta-analyses were conducted using Stata (version 13, StataCorp LP, College Station, TX, USA) using the metan command with input of numerators and denominators for pregnant women and children and the “rr” option to pool the prevalence. We expressed differences between prevalence estimates in pregnant women and children as pooled prevalence ratio (PPR) obtained by meta-analyses using DerSimonian and Laird random-effects models.^17^ We used random effects models because of the wide heterogeneity in study design and to minimise the effect of study size.^18^ The extent of heterogeneity was measured using the *I*^2^, a measure of the proportion of total variability explained by heterogeneity rather than chance expressed as a percentage,^19^ with 0–40% representing no or little heterogeneity, 30–60% moderate heterogeneity, 50–90% substantial heterogeneity, and 75–100% considerable heterogeneity.^20^ To explore determinants of the relation between the prevalence in pregnant women versus children, we examined sources of heterogeneity across studies of the PPR estimates using random-effects meta-regression.^21^ Regression coefficients were presented as odds ratios (ORs) and their corresponding 95% CIs. We estimated between-study variance (τ^2^) using the algorithm of residual (restricted) maximum likelihood, and calculated p values, and 95% CIs for coefficients using the modification by Knapp and Hartung.^22^ For the meta-regression, study-level predictors were considered for inclusion in the initial models if the p value for the univariate association of that variable with the endpoint was <0·2.

We considered the effect of the following predictors: gravidity, study period, location of recruitment for pregnant women (community or antenatal clinic), coverage of antimalarial prevention (chemoprophylaxis or IPTp) in pregnant women, type of diagnostic test, malaria transmission intensity, as defined by the average malaria prevalence among children and pregnant women (as a continuous variable and stratified as <5%, 5–40%, >40%),^23^, ^24^ and ITN coverage. Because there is a high correlation between ITN use in pregnant women and children (appendix), we used data for coverage in children to represent both groups.

HIV infection is known to increase the risk of malaria in pregnancy;^25^ however, unfortunately none of the included studies had a systematic assessment of maternal HIV status. As an approximation of maternal HIV status, we used the information from the prevalence of HIV in women aged 15–49 years in the same study, or data from a Demographic and Health Survey closest to the study date, or data from other sources by country in all people aged 15–49 years (appendix).

We did a sensitivity analysis to explore the potential effect of the type of study included (regional survey versus observational study) and of study quality on the primary outcome by comparing the results of (sub)national surveys with local studies, or results from low-to-moderate studies with those from good quality studies.

### Role of the funding source

The funding institution had no role in the design and development, data extraction, analysis and interpretation of the data, or preparation, review, or approval of the paper. AMvE had full access to all data and had final responsibility for the decision to submit for publication.

## Results

**Figure 1:**
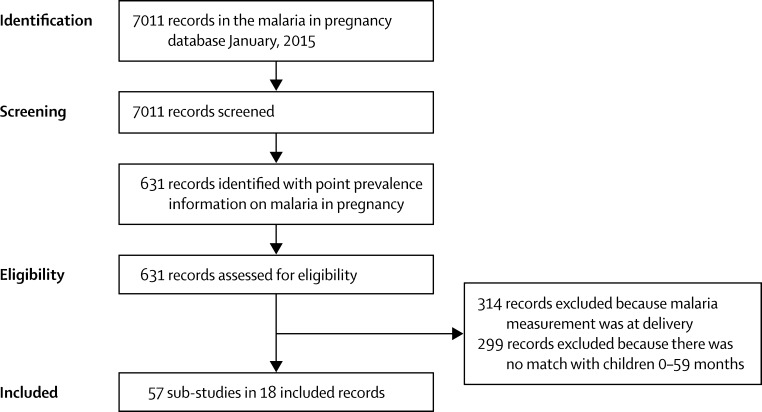
Flow diagram for the literature search

Of 7011 records screened, we identified 18 data sources (13 national or subnational surveys and five local studies)^26^, ^27^, ^28^, ^29^, ^30^, ^31^, ^32^, ^33^, ^34^, ^35^, ^36^, ^37^, ^38^, ^39^, ^40^, ^41^, ^42^, ^43^, ^44^, ^45^, ^46^, ^47^, ^48^, ^49^, ^50^ with information in children that could be matched with studies in pregnant women, resulting in 57 substudies after stratification of information by location and study period (figure 1). Table 1 and the appendix show study characteristics and the results of the quality assessment.

Studies took place between 1983 and 2012; one study recruited participants from an antenatal clinic and all others were from the community.^38^ Five sources used rapid diagnostic malaria tests. There was no uniform reporting method on use of malaria prophylaxis or IPTp in pregnant women; four sources reported case management, and for surveys where IPTp was reported, the use varied from 3% to 94% for at least one dose of sulfadoxine-pyrimethamine. The estimated HIV prevalence in women ranged from 1% to 26%; prevalence was less than 10% in two-thirds of sources (12 of 18). Seven of 18 sources were considered good quality; the least commonly reported criterion was the number of women and children who were missing a blood test result.

**Figure 2:**
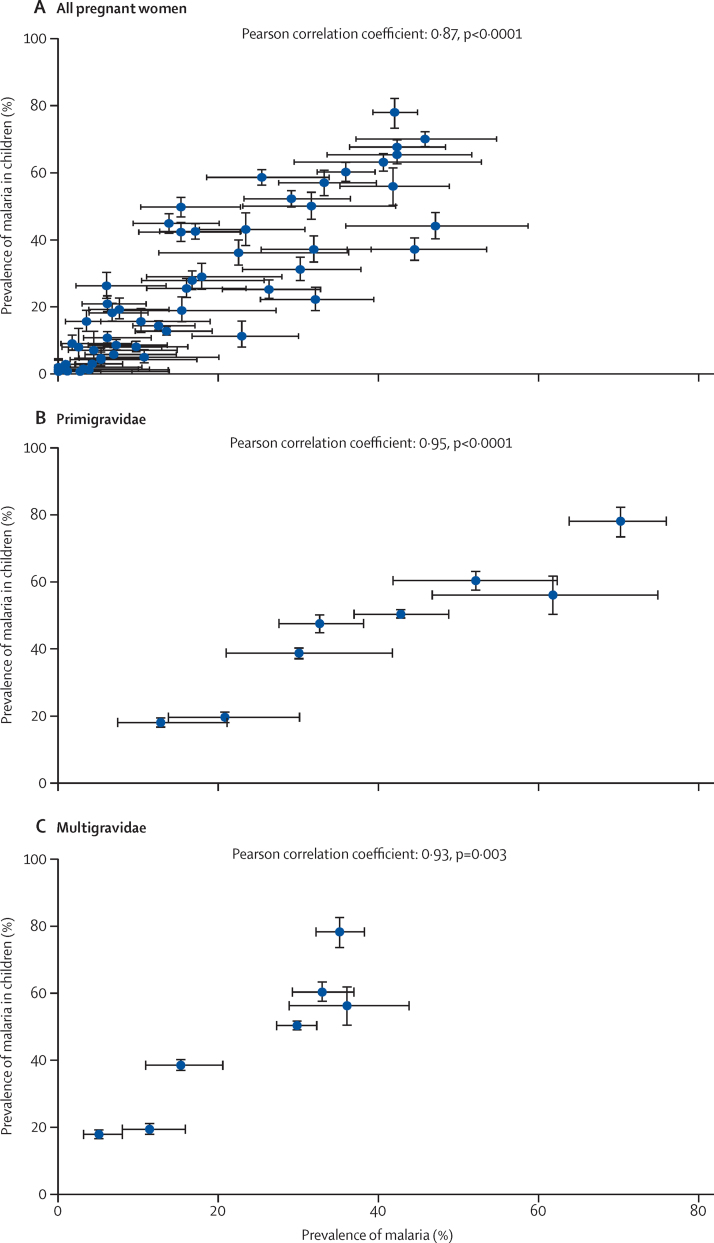
Scatter plots for malaria prevalence in all pregnant women, primigravidae, and multigravidae versus children 0–59 months, sub-Saharan Africa, 1983–2012

**Figure 3:**
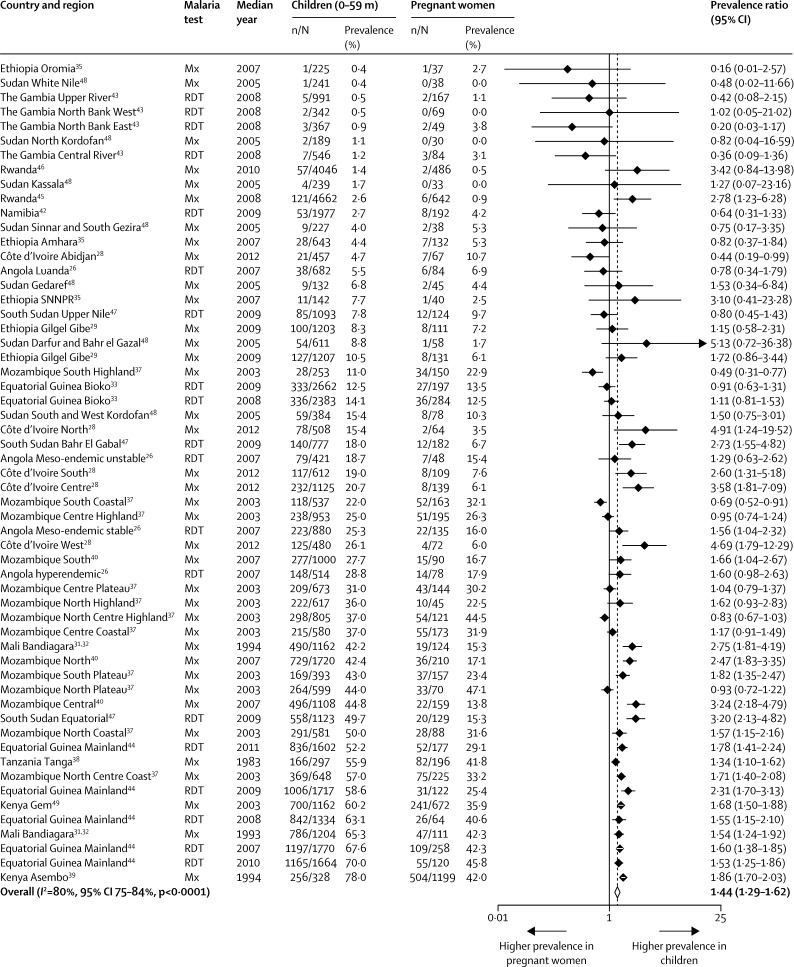
Forest plot of prevalence ratios for malaria in children (0–59 months) versus pregnant women, sub–Saharan Africa, 1983–2012 Mx=microscopy. RDT=rapid diagnostic malaria test. SNNPR=Southern Nations, Nationalities and People's Region. Dotted line shows the pooled prevalence ratio. Studies are listed in ascending order of prevalence of malaria in children.

There was a strong correlation between the prevalence of malaria infection in children aged 0–59 months and pregnant women (Pearson correlation coefficient 0·87, p<0·0001, figure 2), with the average prevalence in children higher than that in pregnant women (PPR 1·44, 95% CI 1·29–1·62, figure 3), but with considerable heterogeneity between studies (*I*^2^=80%, 95% CI 75–84).

Results of meta-regression identified the following effect modifiers of the overall PPR (table 2): higher PPR when the average infection prevalence was higher, and children's age group, with a higher PPR when comparing children aged 6–59 months with pregnant women than when comparing children aged 0–59 months with pregnant women (p=0·017 for the effect of age in the multivariate model).

The type of malaria test used did not have an effect on PPR (rapid diagnostic tests only 1·41, 95% CI 1·18–1·69; microscopy only 1·47, 1·27–1·71; p=0·535 for the effect of diagnostic test in the univariate model).

**Figure 4:**
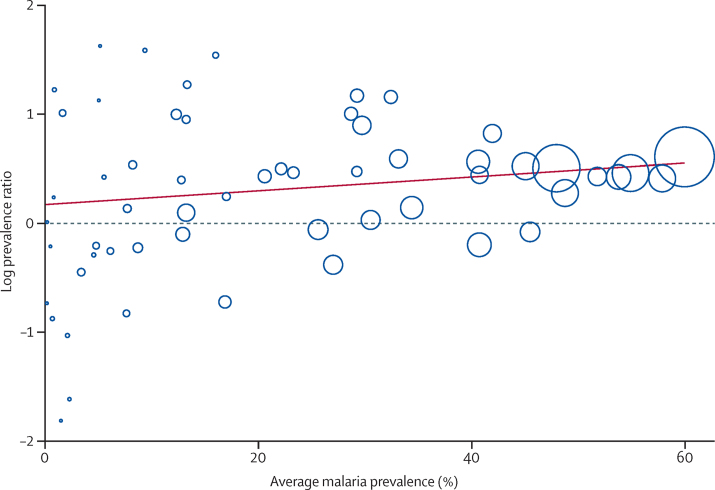
Bubble plot with fitted meta-regression line of the log prevalence ratio: child-maternal malaria prevalence and average malaria prevalence, sub-Saharan Africa, 1983–2012 Circles are sized according to precision of each estimate with larger bubbles for more precise estimates. Average malaria prevalence is the average of malaria prevalence in children and pregnant women.

We explored the relation further for malaria transmission; in subgroup analysis, there was less heterogeneity in areas with a prevalence below 5% (*I*^2^ 42%, 0–70, table 2) but in areas with a higher prevalence *I*^2^ was more than 80%. The graph of the log prevalence ratio (figure 4) showed a more consistent pattern in the areas of high malaria prevalence, but even in areas with a prevalence of over 40%, heterogeneity was high.

A sensitivity analysis in all studies showed that PPRs were lower when analysis was restricted to low-to-moderate quality studies (1·34, 95% CI 1·17–1·54) than when analysis included only higher quality studies (PPR 1·76, 95% CI 1·39–2·24, p=0·086) but this difference in effect was not significant in the multivariate model (p=0·121). PPR for pregnant women versus children also differed slightly when restricting the analysis to local studies only (PPR 1·67, 95% CI 1·46–1·92 compared with subnational or national surveys only, 1·39, 1·21–1·86), but this was not significant (meta-regression: p=0·362).

**Figure 5:**
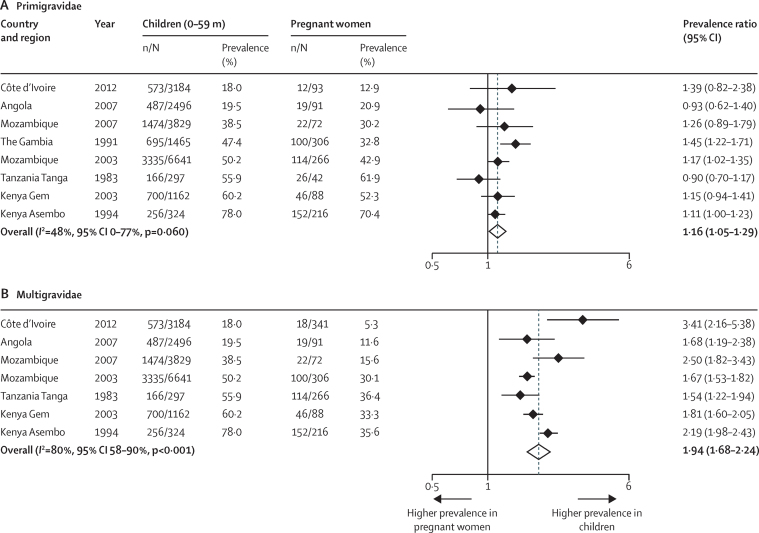
Forest plot of prevalence ratio of malaria in children aged 0–59 months versus primigravidae or multigravidae, sub-Saharan Africa, 1983–2012 Studies are listed in ascending order of prevalence of malaria in children.

A small number of studies provided enough detail to allow analysis by gravidity group.^26^, ^28^, ^37^, ^38^, ^39^, ^40^, ^49^, ^51^ The PPR of children versus primigravidae was much lower (1·16, 95% CI 1·05–1·29, 8 studies, *I*^2^ 48%, figure 5) than the overall PPR, whereas the difference between children and multigravidae was higher (PPR 1·94, 1·68–2·24, 7 studies, *I*^2^ 80%, figure 5). The correlation coefficients were 0·95 (p <0·0001) and 0·93 (p=0·003, figure 2), for the comparison in primigravidae and multigravidae, respectively. All studies were conducted in areas of moderate to high transmission and results of meta-regression did not show a difference in the PPR between children and primigravidae (p=0·992) or multigravidae (p=0·209) when malaria transmission level was taken in to account; however, the number of studies for this analysis was small (table 3 and appendix).

## Discussion

In this meta-analysis we compared the prevalence of malaria infection, as detected by microscopy or rapid diagnostic malaria tests, in pregnant women with the prevalence in children in the same study in the same calendar period and in the same location or region. We showed that the prevalence of malaria infection in pregnant women is lower than that in children aged 0–59 months from the same population, although prevalence estimates in both groups were closely correlated, with a strong linear relation (r=0·87) across the endemicity spectrum.

The difference in prevalence between children and pregnant women was smaller when the pregnant women were primigravidae and also in areas of low malaria transmission. Our findings suggest that changes in malaria infection prevalence in pregnant women attending routine antenatal care may be considered as an alternative indicator to track temporal and spatial trends in malaria transmission intensity.

Antenatal clinic populations are a convenient and easy-to-access group for real-time malaria infection surveillance because most women attend antenatal clinic at least once during pregnancy, even in some hard-to-reach rural areas. Women attend scheduled visits with a focus on preventive health strategies, prompt identification and treatment of illness or conditions, and birth planning. The patterns of malaria prevalence at antenatal booking (that is, before women have received any intervention) may, thus, reflect transmission intensity in their communities.

An advantage of using antenatal clinic data to assess trends in malaria transmission is that in many countries pregnant women are routinely screened for HIV, syphilis, and anaemia at their first antenatal booking visit and the addition of testing for malaria would not require any additional sampling. The large difference in malaria prevalence between primigravidae and multigravidae suggest that gravidity would need to be taken into account.

That the risk of malaria in pregnant women is lower than that in children in areas of moderate-to-high transmission is not surprising. Parasites can sequester in the placenta, avoiding detection by diagnostic tests, and the concomitant peripheral parasite prevalence can be lower than that in the placenta. A meta-analysis by Kattenberg and colleagues^52^ reported a sensitivity of peripheral maternal blood microscopy of 72% (95% CI 62–80) for detection of placental malaria, so if all placental malaria infections had been detected in the peripheral blood, in some regions the prevalence in pregnant women might have approached that recorded in children.

However, in areas of higher malaria transmission the prevalence gap between pregnant women and children increases and the lower detection level in the peripheral blood is not likely to explain the difference. Previous studies and meta-analysis showed that pregnant women with acute malaria are consistently better at clearing parasites after antimalarial treatment with chloroquine or sulfadoxine-pyrimethamine than are children.^10^, ^53^ This finding probably reflects the higher level of acquired protective malarial immunity in pregnant women, especially multigravidae, in areas of high malaria endemicity and, thus, their ability to control and suppress parasite densities when infected relative to the immunity level in young children. Primigravidae generally do not have antibodies to placental-type parasites at the onset of pregnancy, but generate these during the course of pregnancy if exposed to malaria, and some have suggested using these antibody responses as sentinel markers for malaria transmission.^54^

In addition to gravidity, several other factors modified the relation between the population prevalence of malaria infection in pregnant women and children, including the age of the children used for comparison, with greater relative differences with pregnant women in the 6–59 months age group than 0–59 month old children. This likely reflects the lower risk of malaria in the first months of life compared with that later in infancy.^55^

Although there was a good correlation between malaria in children and pregnant women, the high heterogeneity across the malaria spectrum indicates that data in pregnant women may be more useful to assess trends than to use as an approximation of malaria transmission or to estimate malaria prevalence in other vulnerable groups. For example, for a malaria prevalence in pregnant women between 10% and 20% (12 data-points), the prevalence in children varied from 4·7% to 49·7%. The heterogeneity was less in areas of low transmission and in primigravidae.

There are important limitations to this type of secondary analysis that should be considered. First, these data might not be representative of sub-Saharan Africa because the number of studies with available data in both pregnant women and children at the same location and during the same time was small (18 sources). Second, most of the data for the comparison between children and pregnant women came from community-based surveys, and it is not yet clear whether these data are representative of the antenatal population, especially the potential target population for sentinel malaria surveillance––that is, those attending an antenatal clinic for their first booking visit. Most pregnant women in Africa have their first antenatal clinic visit before month 6 of pregnancy (appendix), when the risk of malaria is high, compared with the third trimester (van Eijk, unpublished observation); use of malaria prevention such as chemoprophylaxis or IPTp in women attending for their first antenatal visit is unlikely, so that the prevalence of malaria among first antenatal clinic attendees may be closer to that of children than reflected in our analyses.

However, women who do not attend antenatal clinics may be at greater risk of malaria given that antenatal clinic attendance can be low in some rural populations, and in women with low socioeconomic status; both of these factors have been associated with an increased risk of malaria.^9^, ^16^, ^56^, ^57^ Although this source of selection bias is likely to be small in malaria-endemic Africa where more than 90% women attend an antenatal clinic at least once,^9^ in countries where this is not the case––that is, where more than 10% of women do not attend an antenatal clinic––population-based surveys may be needed to assess whether the risk of malaria infection in these women is different from that in women who do attend antenatal clinics.

In settings where more than 10% of women do not attend ANC, the use of annealing methods should be considered that combine data from a relatively small random community survey sample with the convenience sample obtained from data that can be routinely collected in antenatal clinics, as has been done for HIV studies.^58^ These hybrid prevalence estimators provide more accurate information than those available from using only data derived from antenatal clinics, and are more efficient than when data are collected only through larger (and thus more expensive and only periodic) community-based random survey samples such as in Demographic and Health Surveys or Malaria Indicator Surveys.^58^

Examples of countries with antenatal clinic attendance rates less than 90% in a malarious country include Nigeria (61% in 2013), Mali (74% in 2012–13), Angola (80% in 2006–07), Togo (73% in 2013), and the Central African Republic (68% in 2010) (appendix).

Another limitation of this analysis is that, although average malaria prevalence among children and pregnant women was used for the assessment of malaria endemicity, the 2–9 year age group is typically used for this.^59^ Further, the subnational surveys used a two-stage cluster sampling design and this might have had an effect on the standard error around the prevalence estimate, but we could not take this effect into account in our secondary analysis, which might have resulted in an overestimation of the precision of the effect estimates.

In sensitivity analysis, the PPR from low-to-moderate quality studies was lower than the PPR of higher quality studies. This finding might be partly explained by differences in transmission intensity because the mean prevalence of malaria in children in low-to-moderate studies was about half that observed in the better quality studies (16% *vs* 31%, respectively). An alternative explanation might include different compositions of the study populations in low-to-moderate quality studies, with, for example, more primigravidae or women of young age. However, information available from the included studies was insufficient to explore this theory further.

Although the biology and epidemiology of malaria and HIV differ substantially, lessons can be learned from the extensive experience with the use of antenatal data as a convenience sample for HIV-infection surveillance. For example, the use of hybrid prevalence estimators and the annealing of antenatal data with small random community samples to reduce bias.^58^ Overestimates have been reported when comparing estimates from antenatal clinics with community surveillance: suggested reasons included preferential antenatal attendance (for example, referral of people suspected of having HIV to certain clinics), the geographic under-representation of rural clinics (to obtain the sample size in the required period, high volume antenatal clinics are used which are more likely to be in urban areas), and cultural factors.^60^, ^61^, ^62^, ^63^ However, because of their consistent method and routine collection antenatal clinics are still the main source for trends in countries with generalised epidemics.^63^

Our meta-analysis found a strong linear relation between the prevalence of malaria infection in pregnant women and children from the same population. Routine information on the malaria infection status of pregnant women attending antenatal care might become increasingly available if countries switch from IPTp with sulfadoxine-pyrimethamine to “screen and treat” approaches. This switch could happen because of decreasing malaria transmission rates or increasing high-grade resistance to sulfadoxine-pyrimethamine, the only antimalarial currently recommended for IPTp. Antenatal surveillance for malaria infection, especially during the first antenatal booking visit, should be explored as a pragmatic and sustainable method for the real-time monitoring of malaria trends.

## Figures and Tables

**Table 1:** Characteristics of 18 studies included in the comparison of malaria in children 0–5 years of age versus pregnant women, sub-Saharan Africa, 1983–2012

	**Country and location of recruitment**	**Study period**	**Design**	**Primary objective of study**	Level of information*	**Test and species**	**Sample size**		**Pregnant women: antimalarial for prevention**†	**Children: antimalarial for fever**‡	**ITNs or nets**		**Age**		¶**HIV prevalence estimate (%)**
							Pregnant women	Children			Pregnant Women	Children	Pregnant Women (years)	Children (months)	
Angola MIS 2006–07^26^	Angola, community	2006–07	Survey	Evaluation control malaria	Regional (4)	RDT, Pf	345	2497	SP1+ 3%	7%	ITN 22%	ITN 18%	15–49	6–59	2·0^27^
Côte d’Ivoire DHS 2011–12^28^	Côte d’Ivoire, community	2011–12	Survey	Evaluation control malaria	Regional (5)	Mx, any	451	3184	SP1+ 26%	4%	ITN 40%	ITN 37%	15–49	6–59	4·6^28^
Deribew 2010^29^	Ethiopia, community	2009	Survey	Evaluation ITN use	Local (2)	Mx, any	242	2410	Case management	NR	ITN 63%	ITN 57%	Mean 26	0–59	1·0^30^
Dicko 2003 & Dicko 2005 31, 32	Mali, community	1993–94	Survey	Epidemiology malaria	Local (2)	Mx, any	235	2366	CQ 16%	NR	NR	NR	15–45 (mean 28)	6–59	1·8^27^
Equatorial Guinea MIS 2008 & 2009^33^	Equatorial Guinea Bioko, community	2008, 2009	Survey	Evaluation control malaria	Regional (2)	RDT, Pf	481	5045	SP1+ 46%	6%	ITN 58%	ITN 54%	15–49	0–59	3·9^34^
Graves 2009^35^	Ethiopia, community	2006–07	Survey	Evaluation control malaria	Regional (3)	Mx, any	209	1010	Case management	NR	ITN 19%	ITN 19%	15–49	0–59	1·9^36^
Mabunda 2006^37^	Mozambique, community	2002–03	Survey	Evaluation control malaria	Regional (11)	Mx, Pf	1531	6641	NR	NR	NR	NR	12–44 (mean 26)	0–59	10·7^27^
Matola 1985^38^	Tanzania, ANC and MCH	1983	Survey	Evaluation use of CQ as prevention	Local (1)	Mx, any	196	297	CQ 24%	CQ 11%	NR	NR	16–43 (mean 25)	0–59 mean 14	0·5
McElroy 1999 & Bloland 1999^39^	Kenya, community	1992–96	Cohort	Epidemiology malaria	Local (1)	Mx, any	1047	328	Case management	NR	Net 9%	Net 9%	Mean 26	0–59	25·5^27^
Mozambique MIS 2007^40^	Mozambique, community	2007	Survey	Evaluation malaria control	Regional (3)	Mx, any	459	3828	SP1+ 27%	18%	ITN 7%	ITN 7%	15–49	0–59	13·9^41^
Namibia MIS 2009^42^	Namibia, community	2009	Survey	Evaluation control malaria	National (1)	RDT, Pf	192	1977	SP1+ 8%	6%	ITN 26%	ITN 34%	15–49	6–59	16·4^27^
Nyan 2009 MIS The Gambia^43^	The Gambia, community	2008	Survey	Evaluation control malaria	Regional (5)	RDT, Pf	402	2470	SP1+ 94%	14%	ITN 45%	ITN 43%	15–49	6–59	1·7^27^
Rehman 2013^44^	Equatorial Guinea, community	2007–09	Survey	Evaluation control malaria	Regional (5)	RDT, Pf	741	8087	SP1+ 18-45%	3%	ITN 19%	ITN 19%	NR	12–59	10·0^34^
Rwanda 2007–08 DHS^45^	Rwanda, community	2007–08	Survey	Evaluation control malaria	National (1)	Mx, any	642	4662	SP1+ 51%	1%	ITN 60%	ITN 56%	15–49	6–59	3·7^46^
Rwanda 2010–11 DHS^46^	Rwanda, community	2010–11	Survey	Evaluation control malaria	National (1)	Mx, any	486	4046	Case management	2%	ITN 72%	ITN 70%	15–49	6–59	3·7^46^
South Sudan MIS 2009^47^	South Sudan, community	2009	Survey	Evaluation control malaria	Regional (3)	Mx, any	435	2993	SP1+ 17%	13%	ITN 36%	ITN 25%	15–49 <20, 13%	0–59	3·2^27^
Sudan MIS 2005^48^	Sudan, community	2005	Survey	Evaluation malaria control	Regional (6)	Mx, any	320	2023	CQ or SP 10%	19%	ITN 6%	ITN 8%	15–49	0–59	0·5^27^
Van Eijk 2008^49^	Kenya, community	2003	Survey	Health assessment	Local (1)	Mx, any	672	1162	SP1+ 8%	32%	ITN 69%	ITN 67%	Mean 26	6–59	18·3^50^

IITN=insecticide-treated net; MIS=malaria indicator survey; RDT =rapid diagnostic testing; Pf=*Plasmodium falciparum*; SP1+=at least one dose of sulfadoxine-pyrimethamine. DHS=Demographic and Health Survey; Mx=microscopy; NR= not reported; ANC=antenatal clinic; MCH=maternal and child health clinic.; SP= sulfadoxine-pyrimethamine.

* Level of information: information used at a national level, or by region when it was part of a national or sub-national survey, or at a local level if it was a local study; number of sub-studies derived from the source by location or study period is shown in brackets.

† Prophylaxis as reported in pregnant women, or in women in the same survey who completed a pregnancy in the last 2 or 5 years, for their last pregnancy; for surveys where only SP2+ was reported, SP1+ was estimated as 1·67 × SP2+.^16^

‡ Antimalarial treatment for a fever episode in the previous 2 weeks.

¶ Prevalence in women aged 15–49 years.

**Table 2:** Meta-regression of factors that might affect the prevalence ratio for malaria in children 0–59 months versus pregnant women in sub-Saharan Africa, 1983–2012

		**Number of surveys**	**Pooled prevalence ratio (95% CI)**	***I*^2^ (%) (95% CI) for subgroup analysis**	**Odds ratio meta-regression (95% CI)**	**p value by level**	**τ^2^**	**Variance explained (%)**	**p (overall)**
No covariates		57	1·44 (1·29–1·62)	80 (75–84)			0·182		
Place of recruitment of pregnant women									
	ANC	1	1·34 (1·10–1·62)		0·93 (0·36–2·39)	0·877	0·190	0·0	0·877
	Community	56	1·44 (1·28–1·63)	80 (75–85)	1·00 (Reference)				
Malaria test									
	RDT	19	1·41 (1·18–1·69)	71 (54–82)	0·91 (0·66–1·24)	0·535	0·192	0·0	0·535
	Microscopy	38	1·47 (1·27–1·71)	83 (78–87)	1·00 (Reference)				
Time period (year)									
	<2000	4	1·72 (1·38–2·15)	80 (47–92)	1·26 (0·77–2·07)	0·344	0·175	0·0	0·344
	≥2000	53	1·40 (1·23–1·60)	80 (74–84)	1·00 (Reference)				
Average malaria prevalence * as an indicator of transmission level									
	Continuous	57	..	..	1·00 (0·99–1·02)	0·139	0·177	2·4	0·139
	>40%	13	1·51 (1·33–1·72)	84 (73–90)	1·79 (1·03–3·10)	0·039	0·184	0·0	0·084
	5–40%	31	1·53 (1·24–1·88)	83 (77–88)	1·79 (1·06–3·04)	0·030			
	<5%	13	0·82 (0·47–1·40)	42 (0–70)	1·00 (Reference)				
Antimalarial regimen during pregnancy†									
	None	18	1·17 (0·94–1·46)	89 (84–92)	1·00 (Reference)		0·154	15·5	0·106
	IPTp	29	1·64 (1·41–1·91)	75 (64–82)	1·38 (1·01–1·88)	0·042			
	Prophylaxis‡	10	1·61 (1·27–2·04)	34 (0–68)	1·38 (0·85–2·25)	0·188			
ITN use during pregnancy									
	No ITN information	16	1·27 (1·07–1·51)	90 (86–93)	1·00 (Reference)		0·176	3·0	0·357
	ITN use < 25%	22	1·57 (1·31–1·88)	65 (45–78)	1·18 (0·84–1·68)	0·332			
	ITN use ≥25%	19	1·64 (1·20–2·23)	72 (55–82)	1·29 (0·89–1·88)	0·173			
Age definition of child group									
	0–59 months	31	1·25 (1·07–1·47)	84 (78–88)	1·00 (Reference)		0·156	14·1	0·111
	6–59 months	21	1·67 (1·29–2·18)	73 (58–82)	1·36 (0·98–1·87)	0·063			
	12–59 months	5	1·68 (1·49–1·90)	34 (0–75)	1·38 (0·89–2·14)	0·152			
Estimate of maternal HIV infection†									
	Continuous	57	..	..	0·98 (0·96–1·01)	0·139	0·179	1·6	0·139
	>9%	16	1·40 (1·19–1·65)	89 (83–92)	0·94 (0·69–1·28)	0·676	0·187	0·0	0·676
	≤ 9%	41	1·47 (1·24–1·75)	74 (65–81)	1·00 (Reference)				
Multivariate analysis							0·149	17·9	0·025
Average malaria prevalence as an indicator of transmission level*									
	>40%	17	..	..	2·03 (1·12–3·66)	0·020			
	5–40%	26	..	..	1·97 (1·17–3·31)	0·012			
	<5%	14	..	..	1·00 (Reference)				
Age definition of child group									
	0–59 months	31	..	..	1·00 (Reference)				
	6–59 months	21	..	..	1·49 (1·08–2·07)	0·017			
	12–59 months	5	..	..	1·30 (0·81–2·11)	0·270			

ANC=antenatal clinic. RDT=rapid diagnostic test. IPTp=intermittent preventive treatment in pregnancy. ITN= insecticide treated nets.

* Average malaria prevalence in children and pregnant women.

† Not significant in multivariate analysis.

‡ Any dose for any time period of prophylaxis, not IPTp.

**Table 3:** Subgroup analysis of pooled prevalence ratio of malaria in children versus malaria in pregnant women by gravidity and by average malaria prevalence in children and pregnant women, sub-Saharan Africa, 1983–2012

	**Number of studies**	**Pooled prevalence ratio (95% CI)**	***I*^2^ (%) (95% CI)**	**Odds ratio meta-regression**	**95% CI**	**p**
**Primigravidae**						
>40%	5	1·16 (1·02–1·32)	66 (12–87)	0·99	0·68–1·46	0·992
5%–40%	3	1·16 (0·92–1·47)	0 (0–90)	1·00	Reference	
**Multigravidae**						
>40%	4	1·81 (1·54–2·12)	85 (63–94)	0·77	0·48–1·23	0·209
5%–40%	3	2·38 (1·63–3·48)	69 (0–91)	1·00	Reference	
