# Supplementary material for: Prevalence of malaria infection in pregnant women compared with children for tracking malaria transmission in sub-Saharan Africa: a systematic review and meta-analysis
Source: Lancet Glob Health. 2015 Aug 19;3(10):e617–28. doi: 10.1016/S2214-109X(15)00049-2 (PMC4673091; doi:10.1016/S2214-109X(15)00049-2)
Supplement: Supplementary appendix [file mmc1.pdf]

THE LANCET Global Health

Supplementary appendix

This appendix formed part of the original submission and has been peer reviewed. We post it as supplied by the authors.

Supplement to: van Eijk AM, Hill J, Noor AM, Snow RW, ter Kuile FO. Prevalence of malaria infection in pregnant women compared with children for tracking malaria transmission in sub-Saharan Africa: a systematic review and meta-analysis. *Lancet Glob Health* 2015; published online Aug 19. http://dx.doi.org/10.1016/S2214-109X(15)00049-2.

Supplement

Contents

Supplement S1: Study quality assessment 2

***Table S1*: Quality assessment of 18 sources included in the comparison of malaria in pregnant women *versus* children 0-59 months, sub-Saharan Africa, 1983-2012 .... 3**

Supplement S2: Insecticide treated net us by pregnant women and children 6

***Figure S2:* Scatterplot of use of insecticide nets (%) by pregnant women and children <5 years of age among surveys included in this review, sub Saharan Africa, 2002-2012 .... 6**

Supplement S3: Sources used for an HIV estimate 7

***Figure S3:* Scatterplot of HIV prevalence among women aged 15-49 years (%) versus persons aged 15-49 years (%) (both genders) among 47 national surveys conducted between 2001 and 2014 in sub Saharan Africa 7**

Supplement S4: Metaregression of the prevalence ratio of malaria among children 0-59 months *versus* pregnant women by gravidity 8

***Table S4a:* Meta-regression of factors potentially affecting the prevalence ratio of malaria among children 0-59 months of age *versus* primigravidae, sub-Saharan Africa, 1983-2012 .... 8**

***Table S4b:* Meta-regression of factors potentially affecting the prevalence ratio of malaria among children 0-59 months of age *versus* multigravidae, 7 studies, sub-Saharan Africa, 1983-2012 .... 9**

Supplement S5: Antenatal clinic attendance in malarious countries in sub-Saharan Africa by setting, 2008-2014 10

***Table S5:* Antenatal clinic attendance and timing of first antenatal visit in national surveys in malarious countries by setting and malaria prevalence among children, sub-Saharan Africa, 2008-2014 .... 10**

References 11

Supplement S1: Study quality assessment

Quality assessment

Assessment of these surveys was based on source population, participant selection, completeness of outcome data, and measures to control confounding, after reviewing literature on quality assessment of observational studies.[1,2] Scores were not assigned, but quality was assigned as low-to-moderate or good. A study was deemed to be good if there was an appropriate source population and participant selection (in this case, pregnant women and children selected from the same location at about the same study period), an appropriate malaria test, no missing outcome data, and a description of characteristics of the study population(s), in particular age. Multivariate analysis was not included as a quality criterion because for almost all studies the comparison of malaria in children *versus* pregnant women was not the focus of the manuscript. Outcome was considered 'adequate' if more than 80% of participants initially enrolled were included in the analysis, 'unclear' if the percentage of initially enrolled participants included in the analysis was unclear, and 'inadequate' if less than 80% of those initially enrolled were included in the analysis.

***Table S1*: Quality assessment of 18 sources included in the comparison of malaria in pregnant women *versus* children 0-59 months, sub-Saharan Africa, 1983-2012**

⬢: Adequate or appropriate. ○: Unclear from available information. ▲: Inappropriate or inadaequate reported

Author/year, type of material	Study source population described and adequate	Inclusion/exclusion criteria described and adequate for study purpose	Tests described, appropriate	Incomplete outcome data	Characteristics reported	Other bias and notes	Assessment quality for review
Angola MIS 2006-7[3]<br>Report	Community, both	Yes<br>In random households selected from random clusters for the survey	Yes, rapid diagnostic malaria test	Children 9% missing, PW 0%	Yes, SES, area of residence, education, but not gravidity or age for PW	None detected	Good
	⬢	⬢	⬢	⬢	⬢	⬢	
Côte d'Ivoire DHS 2011-12[4]<br>Report	Community, both	Yes<br>In random households selected from random clusters for the survey	Yes, microscopy	Children 13%<br>PW 21%	Yes, SES, area of residence, education, but not gravidity or age for PW	None detected	Good
	⬢	⬢	⬢	⬢	⬢	⬢	
Deribew 2010[5] Article	Community, both	Yes<br>In villages in study area	Yes, microscopy	Children 12%, NR for PW	Yes, for both	None detected	Low-to-moderate
	⬢	⬢	⬢	▲	⬢	⬢	
Dicko 2003, 2005[6,7]<br>Article	Yes, both from community	Yes, from villages randomly selected	Yes, microscopy	Not reported	Characteristics partly presented (age only for children)	None detected	Low-to-moderate
	⬢	⬢	⬢	▲	⬢	⬢	
Equatorial Guinea MIS Bioko 2008-9[8]<br>Report	Community, both	Yes<br>In random households selected from random clusters for the survey	Yes, rapid diagnostic malaria test	Not reported	Children: yes<br>PW: none	None detected	Low-to-moderate
	⬢	⬢	⬢	▲	⬢	⬢	
Graves 2009[9] Article	Community, both	Yes<br>In random households selected from random clusters for the survey	Yes, microscopy	Not reported	Not reported	Considerable *P. vivax*	Low-to-moderate
	⬢	⬢	⬢	▲	▲	▲	
Mabunda 2006[10]<br>Thesis	Yes, both from community	Yes, from villages randomly selected	Yes, microscopy	Not reported	Yes, age and parity reported for PW	Conducted over two malaria seasons	Low-to-moderate
	⬢	⬢	⬢	▲	⬢	▲	
Matola 1985[11] Article	MCH and ANC clinics of the same two health facilities	Not reported	Yes, microscopy	Not reported	PW: mean age and parity reported. Children: mean age reported	None	Low-to-moderate
	⬢	▲	⬢	▲	⬢	⬢	
McElroy/Bloland 1999[12,13]<br>Article	Community, both	Yes, in study cohort	Yes, microscopy	Not reported	Yes	None detected	Low-to-moderate
	⬢	⬢	⬢	▲	⬢	⬢	

Author/year, type of material	Study source population described and adequate	Inclusion/exclusion criteria described and adequate for study purpose	Tests described, appropriate	Incomplete outcome data	Characteristics reported	Other bias and notes	Assessment quality for review
Mozambique MIS 2007[14]<br>Report	Community, both	Yes<br>In random households selected from random clusters for the survey	Yes, microscopy	Not reported	Yes, SES, area of residence, education, but no gravidity, age for PW	None detected	Low-to-moderate
	●	●	●	▲	●	●	
Namibia MIS 2009[15]<br>Report	Community, both	Yes<br>In random households selected from random clusters for the survey	Yes, rapid diagnostic malaria test	5% for children, 2% for pregnant women	No	None detected	Low-to-moderate
	●	●	●	●	▲	●	
Nyan 2009[16]<br>Report	Community, both	Yes<br>In random households selected from random clusters for the survey	Yes, rapid diagnostic malaria test	Not reported	Yes, for both	None detected	Low-to-moderate
	●	●	●	▲	●	●	
Rehman 2013[17]<br>Article	Community, both	Yes<br>In random households selected from random clusters for the survey	Yes, rapid diagnostic malaria test	Children 7%<br>PW 42%	Children: age<br>PW: none	None detected	Low-to-moderate
	●	●	●	▲	▲	●	
Rwanda DHS 2007-8[18]<br>Report	Community, both	Yes<br>In random households selected from random clusters for the survey	Yes, microscopy	Children 14%<br>PW 5%	Yes, SES, area of residence, education, but no gravidity, age for PW	None detected	Good
	●	●	●	●	●	●	
Rwanda DHS 2010-11[19]<br>Report	Community, both	Yes<br>In random households selected from random clusters for the survey	Yes, microscopy	Not reported	Yes, SES, area of residence, education, but no gravidity, age for PW	None detected	Low-to-moderate
	●	●	●	▲	●	●	
South Sudan MIS 2009[20]<br>Report	Community, both	Yes<br>In random households selected from random clusters for the survey	Yes, rapid diagnostic malaria test	Children 3%<br>PW 17%	Yes, SES, area of residence, education, age but no gravidity for PW	None detected	Good
	●	●	●	●	●	●	
Sudan MIS 2005[21]<br>Report	Community, both	Yes<br>In random households selected from random clusters for the survey	Yes, microscopy	Overall blood samples 7% missing	Yes, area of residence but no other characteristics	None detected	Good
	●	●	●	●	●	●	
Van Eijk 2008[22]<br>Article	Yes, both from community	Yes, random selection for children, all pregnancies for PW in study area	Yes, microscopy	Children and PW <1%	Characteristics present for both	None detected	Good

Author/year, type of material	Study source population described and adequate	Inclusion/exclusion criteria described and adequate for study purpose	Tests described, appropriate	Incomplete outcome data	Characteristics reported	Other bias and notes	Assessment quality for review
	●	●	●	●	●	●	

Abbreviations (in alphabetical order): ANC: antenatal clinic, DHS: Demographic and Health survey, MCH: Mother and Child Health Clinic, MIS: malaria indicator survey, NR: Not reported, PW: pregnant women, SES: socio-economic status

Supplement S2: Insecticide treated net us by pregnant women and children

Figure S2: **Scatterplot of use of insecticide nets (%) by pregnant women and children <5 years of age among surveys included in this review, sub Saharan Africa, 2002-2012**

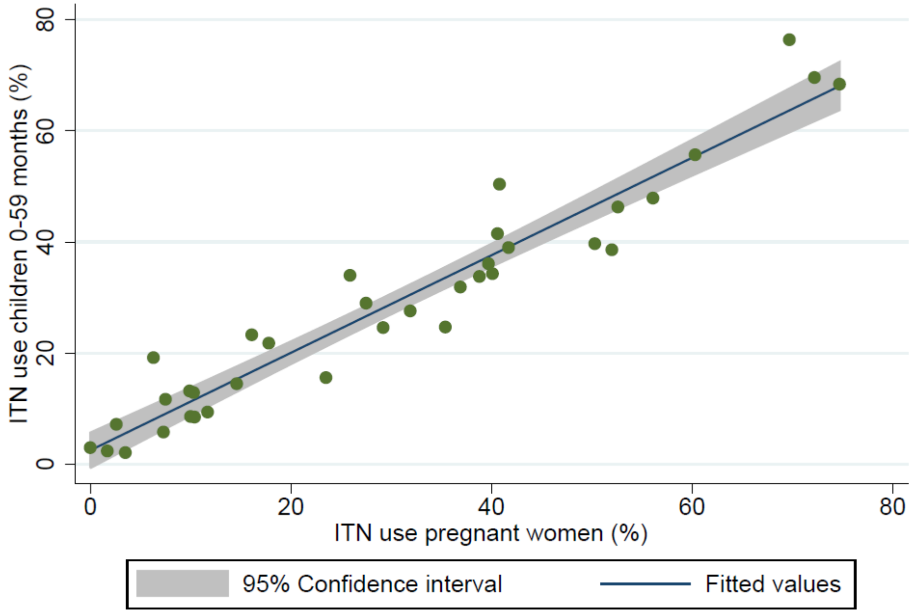

ITN: Insecticide treated net; ITN use defined as use of an insecticide treated net the night before the survey

Pearson correlation coefficient 0·96

Supplement S3: Sources used for an HIV estimate

Estimate HIV

If the malaria information was retrieved from a Demographic and Health survey (DHS) or an AIDS Indicator Survey (AIS) where HIV testing was conducted, results for HIV were obtained from the same report for women aged 15-49 years. If another source was used, results for HIV were obtained from a DHS or AIS report close in date (within 5 years). If this was not available, HIV estimates for the age group 15-49 years of age were obtained for the year involved from Index mundi and these were multiplied by 1·2 to obtain the estimate among women aged 15-49 years.[23] The multiplication factor was obtained after comparing HIV prevalence by gender in 47 African DHS reports with information on the overall HIV prevalence. For Mozambique, the prevalence by region (North, Central and South) was proportionally adjusted using the distribution of HIV among women reported in the AIS of 2009.[10,24] A study in Tanzania conducted at the start of the HIV-epidemic was assigned an arbitrary value of 0·5%.[11] In Kenya, women in Nyanza province were 2·6 times more likely to have HIV compared to the national estimate (both men and women), and this was taken into account for the HIV estimate for Kenya in 1992-1996.[13,25]

Figure S3: **Scatterplot of HIV prevalence among women aged 15-49 years (%) versus persons aged 15-49 years (%) (both genders) among 47 national surveys conducted between 2001 and 2014 in sub Saharan Africa**

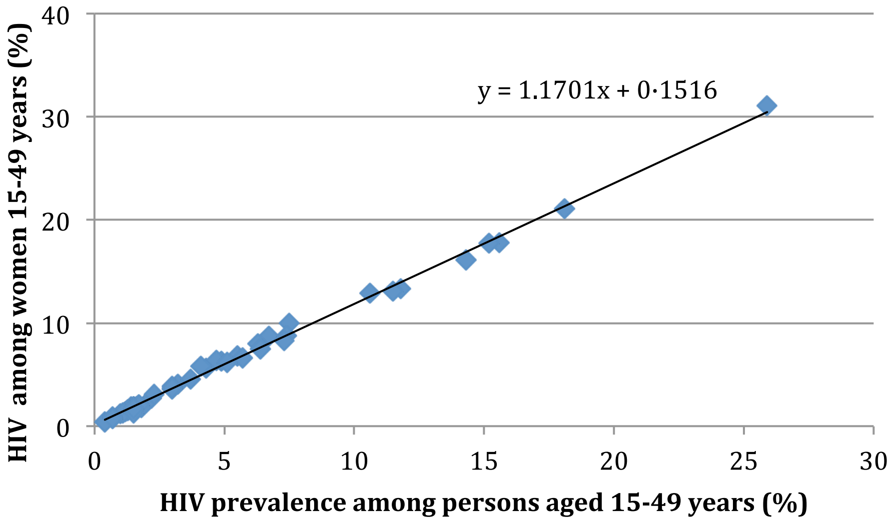

Supplement S4: Metaregression of the prevalence ratio of malaria among children 0-59 months *versus* pregnant women by gravidity

Table S4a: **Meta-regression of factors potentially affecting the prevalence ratio of malaria among children 0-59 months of age *versus* primigravidae, sub-Saharan Africa, 1983-2012**

	**Univariate analysis**									
**Covariate**	**Type of covariate**	**No. of surveys†**	**Pooled prevalence ratio by subgroup**	**I^2 and 95% CI for subgroup analysis (%)**	**Odds ratio meta-regression**	**95% CI**	**p-value by level**	**τ^2**	**Variance explained (%)**	**p-value overall**
No covariates		8						0·010		
(Sub)national survey	Yes	4	1·17, 1·04-1·32	0, 0-85	1·01	0·75-1·37	0·939	0·014	0·0	0·939
	No	4	1·15, 0·97-1·37	76, 33-91	1·00	Reference				
Place of recruitment pregnant women	ANC	1	0·90, 0·70-1·17		0·76	0·51-1·12	0·130	0·006	40·0	0·130
	Community	7	1·20, 1·08-1·32	36, 0-73	1·00	Reference				
Malaria test	RDT	1	0·94, 0·62-1·40		0·79	0·44-1·42	0·368	0·010	0·0	0·368
	BS	7	1·18, 1·05-1·31	53, 0-80	1·00	Reference				
Time period	2000+	5	1·16, 1·05-1·29	0, 0-79	1·00	Reference	0·964	0·015	0·0	0·964
	<2000	3	1·15, 0·90-1·47	84, 53-95	0·99	0·74-1·34				
Malaria transmission intensity*	Continuous	8			0·99	0·99-1·01	0·582	0·011	0·0	0·582
	>40%	5	1·16, 0·92-1·47	66, 12-87	0·99	0·68-1·46	0·992	0·012	0·0	0·992
	5-40%	3	1·16, 1·02-1·32	0, 0-90	1·00	Reference				
Antimalarial regimen during pregnancy	None	2	1·13, 1·04-1·23		1·00	Reference		0·020	0·0	0·971
	IPTp	4	1·16, 0·99-1·35	0, 0-85	1·02	0·68-1·52	0·915			
	Prophylaxis†	2	1·15, 0·72-1·86		1·04	0·68-1·59	0·819			
ITN use during Pregnancy	No ITN information	5	1·16, 1·02-1·32	66, 12-87	1·00	Reference		0·013	0·0	0·807
	ITN use < 25%	2	1·11, 0·83-1·48		0·95	0·60-1·51	0·790			
	ITN use ≥25%	1	1·39, 0·82-2·38		1·20	0·53-2·71	0·585			
Age definition of child group	0-59 months	4	1·11, 1·03-1·20	7, 0-86	1·00	Reference		0·0	100·0	0·096
	6-59 months	3	1·16, 0·92-1·47	0, 0-90	1·04	0·75-1·45	0·751			
	Other	1	1·45, 1·22-1·71		1·30	1·02-1·66	0·039			
Estimate maternal HIV infection (%)	Continuous	8			1·00	0·98-1·01	0·861	0·014	0·0	0·861
	>9%	4	1·14, 1·06-1·23	0, 0-85	0·99	0·73-1·33	0·939	0·014	0·0	0·939
	<= 9%	4	1·14, 0·85-1·54	74, 27-91	1·00	Reference				
Quality survey	Good	5	1·17, 1·02-1·35	67, 15-87	1·04	0·74-1·45	0·792	0·013	0·0	0·792
	Low to moderate	3	1·13, 0·95-1·35	0, 0-90	1·00	Reference				

Abbreviations: ANC: antenatal clinic, CI: confidence interval, ITN: insecticide treated net, RDT: rapid diagnostic malaria test, SP: sulfadoxine-pyrimethamine

Note: None of the factors with a p-value <0·2 (age definition of child, place of recruitment or maternal HIV infection) was significant in multivariate analysis

* Average malaria transmission: average of child and maternal malaria

†Any dose for any time period of prophylaxis, i.e. not IPTp regimen

Table S4b: **Meta-regression of factors potentially affecting the prevalence ratio of malaria among children 0-59 months of age *versus* multigravidae, 7 studies, sub-Saharan Africa, 1983-2012**

	**Univariate analysis**									
**Covariate**	**Type of covariate**	**No. of surveys†**	**Pooled prevalence ratio by subgroup**	**I^2 and 95% CI for subgroup analysis (%)**	**Odds ratio meta-regression**	**95% CI**	**p-value by level**	**τ^2**	**Variance explained (%)**	**p-value overall**
No covariates		7	1·94, 1·68-2·24					0·032		
(Sub)national survey	Yes	4	2·12, 1·56-2·88	80, 46-92	1·13	0·68-1·88	0·567	0·047	0·0	0·567
	No	3	1·86, 1·53-2·27	82, 46-94	1·00	Reference				
Place of recruitment pregnant women	ANC	1	1·54, 1·22-1·94		0·76	0·39-1·46	0·328	0·032	1·7	0·328
	Community	6	2·02, 1·72-2·36	81, 60-91	1·00	Reference				
Malaria test	RDT	1	1·68, 1·19-2·38		0·84	0·37-1·90	0·614	0·041	0·0	0·614
	Microscopy	6	1·97, 1·69-2·31	83, 64-92	1·00	Reference				
Time period	2000+	5	1·96, 1·65-2·33	73, 32-89	1·00	Reference	0·744	0·050	0·0	0·744
	<2000	2	1·86, 1·30-2·67	88, 52-97	0·93	0·53-1·63				
Malaria transmission intensity*	Continuous				0·99	0·98-1·01	0·420	0·042	0·0	0·420
	>40%	4	1·81, 1·54-2·12	85, 63-94	0·77	0·48-1·23	0·209	0·025	21·1	0·209
	5-40%	3	2·38, 1·63-3·48	69, 0-91	1·00	Reference				
Antimalarial regimen during pregnancy	None	2	1·91, 1·44-2·54	94, 83-98	1·00	Reference		0·048	0·0	0·567
	IPTp	4	2·16, 1·64-2·85	73, 23-90	1·13	0·61-2·07	0·615			
	Prophylaxis†	1	1·54, 1·22-1·94		0·80	0·34-1·91	0·523			
ITN use during Pregnancy	No ITN information	4	1·81, 1·54-2·12	85, 63-94	1·00	Reference		0·020	37·4	0·202
	ITN use < 25%	2	2·06, 1·40-3·04	63, 0-92	1·15	0·69-1·89	0·495			
	ITN use ≥25%	1	3·41, 2·16-5·38		1·89	0·83-4·26	0·097			
Age definition of child group	0-59 months	4	1·81, 1·54-2·12	85, 63-94	1·00	Reference		0·025	21·1	0·209
	6-59 months	3	2·38, 1·63-3·48	69, 0-91	1·30	0·81-2·08	0·209			
	12-59 months	0								
Estimate maternal HIV infection (%)	Continuous	7			1·01	0·98-1·04	0·651	0·039	0·0	0·651
	>9%	4	1·96, 1·66-2·31	85, 63-94	1·02	0·59-1·78	0·923	0·044	0·0	0·923
	<= 9%	3	2·00, 1·28-3·13	81, 42-94	1·00	Reference				
Quality survey	Good	4	1·91, 1·56-2·34	87, 68-95	0·93	0·54-1·60	0·754	0·047	0·0	0·754
	Low to moderate	3	2·08, 1·48-2·92	74, 14-92	1·00	Reference				

Abbreviations: ANC: antenatal clinic, CI: confidence interval, IPTp: intermittent preventive treatment in pregnancy, ITN: insecticide treated net, RDT: rapid diagnostic malaria test,
* Average malaria transmission: average of child and maternal malaria
†Any dose for any time period of prophylaxis, not IPTp regimen

Supplement S5: Antenatal clinic attendance in malarious countries in sub-Saharan Africa by setting, 2008-2014

***Table S5:* Antenatal clinic attendance and timing of first antenatal visit in national surveys in malarious countries by setting and malaria prevalence among children, sub-Saharan Africa, 2008-2014**

Country	Survey	At least one antenatal visit, %			Start of 1st visit before 6 months, %			Survey	Malaria prevalence in survey among children months, %				
		All	Urban	Rural	All	Urban	Rural		Test	Age*	All	Urban	Rural
Angola	NA							MIS 2011	Mx	6-59	10·1	1·4	14·0
Benin	DHS 2011-12	85·8	91·3	82·1	75·6	79·6	72·8	DHS 2011-12	Mx	6-59	28·4	20·8	33·2
Burkina Faso	DHS 2010	94·9	98·3	94·1	80·2	87·1	78·6	DHS 2010	Mx	6-59	65·9	29·8	72·9
Burundi	DHS 2010	98·9	99·0	98·9	64·7	80·4	63·2	DHS 2010	ND				
Cameroon	DHS 2011	84·7	95·6	75·6	69·9	82·5	59·4	DHS 2011	RDT	6-59	30·0	20·6	37·1
CAR	MICS 2010	68·2	88·3	57·4	NA				NA				
Chad	MICS 2010	53·2	78·3	45·7	NA				NA				
Republic of Congo	DHS 2011-12	92·6	95·9	86·8	84·5	88·7	76·8	DHS 2011-12	ND				
DR Congo	DHS 2013-14	88·4	94·1	85·8	57·7	68·0	52·9	DHS 2013-14	Mx	6-59	22·6	19·6	23·9
Côte d'Ivoire	DHS 2011-12	90·6	96·7	86·5	64·3	77·2	55·9	DHS 2011-12	MX	6-59	18·0	7·4	24·1
Equatorial Guinea	DHS 2011	91·3	93·3	89·4	87·4	90·0	84·9	DHS 2011	RDT	6-59	47·8	29·9	62·6
Eritrea	DHS 2010	88·5	97·3	84·2	70·7	83·6	64·5		ND				
Ethiopia	DHS 2011	33·9	76·0	26·4	27·7	62·5	21·5		ND				
Gabon	DHS 2012	94·7	96·1	85·7	88·9	90·8	77·5		ND				
The Gambia	DHS 2013	86·2	87·5	84·9	NA			DHS 2013	Mx	6-59	0·8	1·1	0·5
Ghana	MICS 2011	96·4	98·0	95·2	NA			MICS 2011	Mx	6-59	27·5	13·1	38·8
Guinea	DHS 2012	85·2	96·3	80·8	70·8	85·8	64·8	DHS 2012	Mx	6-59	43·9	18·1	52·6
Kenya	DHS 2014	95·5	97·8	94·0	NA			MIS 2010	Mx	6-59	8·2	NA	NA
Liberia	DHS 2013	95·9	98·0	93·4	93·3	90·7	87·5		ND				
Madagascar	DHS 2008-09	86·3	94·7	85·1	69·6	85·4	67·3	MIS 2013	Mx	6-59	9·1	2·9	9·5
Malawi	DHS 2010	94·7	96·2	94·4	60·6	61·7	60·5	MIS 2012	Mx	6-59	27·7	9·4	30·4
Mali	DHS 2012-13	74·2	93·2	69·3	60·7	82·0	55·1	DHS 2012-13	Mx	6-59	51·6	16·8	59·5
Mauritania	MICS 2011	84·2	92·4	78·5	NA				NA				
Mozambique	DHS 2011	90·6	96·3	88·2	60·0	65·6	57·7	DHS 2011	Mx	6-59	35·1	16·2	42·2
Namibia	DHS 2013	96·6	96·7	96·5	80·9	80·7	81·1	DHS 2013	ND				
Niger	DHS 2012	82·8	96·9	80·4	62·0	84·5	58·2	DHS 2012	ND				
Nigeria	DHS 2013	60·6	86·0	46·5	47·5	65·8	37·5	MIS 2010	Mx	6-59	42·0	22·5	47·7
Rwanda	DHS 2010	98·0	98·3	98·0	76·5	78·5	76·3	DHS 2010	Mx	6-59	1·4	0·8	1·4
Sao Tome and Principe	DHS 2008-09	97·9	98·4	97·5	81·7	84·0	79·6		ND				
Senegal	DHS 2014	96·2	99·2	93·8	85·9	91·5	81·3	DHS 2014	Mx	6-59	1·2	0·3	1·9
Sierra Leone	DHS 2013	97·1	98·2	96·7	86·5	86·6	86·4	MIS 2013	Mx	6-59	42·9	28·1	48·0
Somalia	MICS 2011	28·0	38·2	15·3	NA				NA				
South Sudan	MICS 2010	40·3	57·6	34·2	NA				NA				
Sudan	MICS 2010	74·3	84·3	70·4	NA				NA				
Tanzania	DHS 2010	95·9	98·6	95·1	64·8	64·7	66·1	MIS 2011-12	Mx	6-59	4·1	1·0	4·7
Togo	DHS 2013	72·7	96·5	58·4	69·2	80·4	63·3		Mx	6-59	36·4	14·5	47·4
Uganda	DHS 2011	94·9	97·4	94·4	64·7	68·8	63·9	MIS 2009	Mx	0-59	42·4	15·3	47·1
Zambia	DHS 2013-14	95·7	98·6	94·0	79·6	79·3	79·8	MIS 2012	Mx	0-59	14·9	3·7	20·2
Zimbabwe	DHS 2011-12	89·8	89·9	89·8	58·9	54·5	60·9		ND				

Abbreviations: CAR: Central African Republic, DHS: Demographic and Health Survey, DRC: Democratic Republic of the Congo, MICS: Multiple Indicator Cluster Survey, MIS: Malaria Indicator Survey, Mx: microscopy, NA: not available, ND: not done, RDT: rapid diagnostic malaria test

*Age in months

References

1. von Elm E, Altman DG, Egger M, et al. The Strengthening the Reporting of Observational Studies in Epidemiology (STROBE) statement: guidelines for reporting observational studies. *Bull World Health Organ* 2007; **85**: 867–72.
2. Sanderson S, Tatt ID, Higgins JP. Tools for assessing quality and susceptibility to bias in observational studies in epidemiology: a systematic review and annotated bibliography. *Int J Epidemiol* 2007; **36**: 666–76.
3. Consultaria de Servicos e Pesquisas Lda., Consultario de Gestao e Administracao em Saude-Consaude Lda., Macro International Inc. Angola Malaria Indicator Survey 2006-07. Calverton: COSEP Lda., Consaude LDA., and Macro International Inc., 2007.
4. Institut National de la Statistique and ICF International. Côte d'Ivoire Demographic and Health Survey and Multiple Indicator Survey 2011-2012. Calverton: INS and ICF International, 2012.
5. Deribew A, Alemseged F, Birhanu Z, et al. Effect of training on the use of long-lasting insecticide-treated bed nets on the burden of malaria among vulnerable groups, south-west Ethiopia: baseline results of a cluster randomized trial. *Malar J* 2010; **9**: 121.
6. Dicko A, Mantel C, Kouriba B, et al. Season, fever prevalence and pyrogenic threshold for malaria disease definition in an endemic area of Mali. *Trop Med Int Health* 2005; **10**: 550–6.
7. Dicko A, Mantel C, Thera MA, et al. Risk factors for malaria infection and anemia for pregnant women in the Sahel area of Bandiagara, Mali. *Acta Trop* 2003; **89**: 17–23.
8. Ministry of Health and Social Welfare, Medical Care Development International. Equatorial Guinea Malaria Control Initiative (EGMCI) Annual Malaria Indicator Survey Report 2007-2009, 2009.
9. Graves PM, Richards FO, Ngondi J, et al. Individual, household and environmental risk factors for malaria infection in Amhara, Oromia and SNNP regions of Ethiopia. *Trans R Soc Trop Med Hyg* 2009; **103**: 1211–20.
10. Mabunda SJA. The epidemiology and the burden of malaria in Mozambique: University of Barcelona, Faculty of Medicine, Department of Public Health, Barcelona, Spain; 2006.
11. Matola YG, Malle LN. Factors affecting the compliance of malaria chemosuppression with chloroquine at some maternal and child health clinics in Tanga Region, Tanzania. *East Afr Med J* 1985; **62**: 720–4.
12. Bloland PB, Boriga DA, Ruebush TK, et al. Longitudinal cohort study of the epidemiology of malaria infections in an area of intense malaria transmission II. Descriptive epidemiology of malaria infection and disease among children. *Am J Trop Med Hyg* 1999; **60**: 641–8.
13. McElroy PD, Lal AA, Hawley WA, et al. Analysis of repeated hemoglobin measures in full-term, normal birth weight Kenyan children between birth and four years of age. III. The Asemobo Bay Cohort Project. *Am J Trop Med Hyg* 1999; **61**: 932–40.
14. Ministry of Health Mozambique, National Directorate of Public Health, National Malaria Control Programme. National Malaria Indicator Survey Mozambique (MIS - 2007). 2009.
15. Ministry of Health and Social Services Namibia. Namibia Malaria Indicator Survey 2009. Windhoek: National Vector-borne Diseases Control Programme, 2009.
16. Nyan O, Jallow COA, Manneh K, Jarjou E. Malaria baseline survey Final report (The Gambia). The Gambia, 2009.
17. Rehman AM, Mann AG, Schwabe C, et al. Five years of malaria control in the continental region, Equatorial Guinea. *Malar J* 2013; **12**: 154.
18. Ministry of Health of Rwanda, National Institute of Statistics of Rwanda, ICF Macro. Rwanda Interim Demographic and Health Survey 2007-08. Calverton: Minisanté, INSR and ICF Macro, 2009.
19. National Institute of Statistics of Rwanda, Ministry of Health Rwanda, ICF International. Rwanda Demographic and Health Survey 2010. Calverton: NISR, MOH and ICF International, 2012.
20. Eyobo MB, Awur AC, Wani G, et al. Malaria indicator survey 2009, South Sudan: baseline results at household level. *Malar J* 2014; **13**: 45.
21. National Malaria Control Program Sudan. Malaria prevalence and coverage indicators survey Sudan October 2005. Khartoum: Federal Ministry of Health (Sudan), National Malaria Control Program, 2005.
22. van Eijk AM, Lindblade KA, Odhiambo F, et al. Reproductive health issues in rural Western Kenya. *Reprod Health* 2008; **5**: 1.
23. Index Mundi. Country Facts. http://www.indexmundi.com/ (accessed November 25, 2014).
24. National Institute of Health Mozambique, National Institute of Statistics, Macro I. HIV/AIDS sero-behavioural survey in Mozambique 2009. Calverton: National Institute of Health, National Institute of Statistics, ICF Macro, 2010.
25. Central Bureau of Statistics Kenya, Ministry of Health of Kenya, ORC Macro. Kenya Demographic and Health Survey 2003. Calverton: CBS, MOH, and ORC Macro, 2004.
